# Strengthening Chronic Disease Prevention Programming: The Toward Evidence-Informed Practice (TEIP) Program Evidence Tool

**DOI:** 10.5888/pcd10.120107

**Published:** 2013-05-30

**Authors:** Dayna Albert, Rebecca Fortin, Christine Herrera, Barbara Riley, Rhona Hanning, Anne Lessio, Brian Rush

**Affiliations:** Author Affiliations: Dayna Albert, Rebecca Fortin, Anne Lessio, formerly Ontario Public Health Association, Toronto, Ontario; Christine Herrera, University of Toronto, formerly Ontario Public Health Association, Toronto, Ontario; Rhona Hanning, University of Waterloo, Waterloo, Ontario; Brian Rush, University of Toronto, Toronto, Ontario.

## Abstract

In public health and chronic disease prevention there is increasing priority for effective use of evidence in practice. In Ontario, Canada, despite various models being advanced, public health practitioners are seeking ways to identify and apply evidence in their work in practical and meaningful ways. In a companion article, “Strengthening Chronic Disease Prevention Programming: The Toward Evidence-Informed Practice (TEIP) Program Assessment Tool,” we describe use of a tool to assess and strengthen program planning and implementation processes using 19 criteria derived from best and promising practices literature. In this article, we describe use of a complementary Program Evidence Tool to identify, synthesize, and apply a range of evidence sources to strengthen the content of chronic disease prevention programming.

The Program Evidence Tool adapts tools of evidence-based medicine to the unique contexts of community-based health promotion and chronic disease prevention. Knowledge management tools and a guided dialogue process known as an Evidence Forum enable community stakeholders to make appropriate use of evidence in diverse social, political, and structural contexts. Practical guidelines and worksheets direct users through 5 steps: 1) define an evidence question, 2) develop a search strategy, 3) collect and synthesize evidence, 4) interpret and adapt evidence, and 5) implement and evaluate. We describe the Program Evidence Tool’s benefits, strengths, challenges, and what was learned from its application in 4 Ontario public health departments. The Program Evidence Tool contributes to the development and understanding of the complex use of evidence in community-based chronic disease prevention.

## Background

The ability to understand and incorporate evidence into practice is a public health core competency in Ontario (1), Canada (2), the United States (3), and elsewhere (4) and is considered a key component of public health training (5). Nonetheless, it remains difficult to achieve and is inadequately supported by existing tools and processes (6).

Several approaches have been used to facilitate use of evidence, such as organizational diagnostic tools (7), measures of knowledge exchange outcomes (8), online tool kits and guides (9,10), and critically appraised evidence collections such as the *Guide to Community Preventive Services* (www.thecommunityguide.org) in the United States, and Health-evidence.ca (www.health-evidence.ca) and the Canadian Best Practices Portal in Health Promotion and Chronic Disease Prevention (http://cbpp-pcpe.phac-aspc.gc.ca/) in Canada.

Despite these supports, community-based practitioners face multiple challenges using evidence. For example, relevant evidence may not be available or readily accessible; skills, time and resources may be lacking; and organizational infrastructure may not facilitate knowledge exchange (11,12). Adapting interventions to the local context (11,13) remains a challenge despite the use of adaptation guidelines (14,15), and very few practical, context-sensitive evaluations are available in the published literature (16). As a result, there is a need to look at broader definitions of evidence, including evaluations, prior experience, and stakeholder opinion (17).

Toward Evidence-Informed Practice (TEIP) is a set of tools developed at the Ontario Public Health Association to facilitate the use of relevant evidence in community-based health promotion and chronic disease prevention. TEIP tools recognize the high level of engagement required among researchers, decision makers, and local stakeholders, as well as the need to build supports and overcome organizational barriers to facilitate the use of evidence.

A Program Assessment Tool has been developed to identify strengths and areas for improvement in the planning and implementation of local chronic disease prevention programs (18). A review of 22 community-level program assessments showed that most interventions were not informed by relevant theory and research. Practitioners expressed the need for a step-by-step resource that would assist them in identifying and using evidence for program planning. The result was the Program Evidence Tool.

## The Program Evidence Tool

The Program Evidence Tool consists of a set of guidelines and worksheets that provide step-by-step support in identifying and applying relevant sources of evidence to strengthen local chronic disease prevention programming. The tool aims to balance scientific rigor with the needs and challenges of evidence use at the local level. Existing models of evidence use (6,10,19–24) were reviewed, and an advisory committee consisting of public health practitioners and knowledge-exchange researchers was formed.

The Program Evidence Tool (available upon request from the authors) consists of 5 steps: 1) define evidence question, 2) develop search strategy, 3) collect and synthesize evidence, 4) interpret and adapt evidence, and 5) implement and evaluate.

### Step 1: Define evidence question

**Figure 1:**
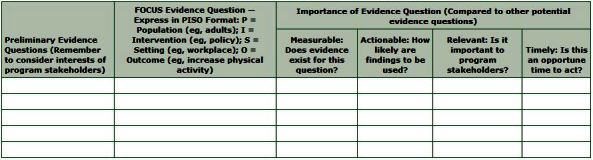
The evidence collection worksheet is used to develop and select the priority evidence question by focusing and establishing the importance of potential evidence questions.

The search for evidence begins with defining the evidence question. A well-defined question addresses a priority issue and keeps the search focused and manageable. Brainstorming potential evidence questions with program stakeholders facilitates the identification of relevant questions. Expressing the evidence question in PISO (Population, Intervention [or approach], Setting, and Desired Outcomes) format focuses the evidence question and identifies useful search terms. Finally, comparing potential evidence questions in 4 areas — measurability, actionability, relevance, and timeliness — facilitates selection of the priority evidence question. The evidence question worksheet (Figure 1) guides and tracks the process of brainstorming, focusing, and prioritizing evidence questions.

### Step 2: Develop search strategy

Without a map to guide the search for evidence, one can easily become lost in search engines, crisscrossing online and print journals and skipping from one reference list to the next. A search strategy sets out specific sites to be searched. Once sites are established, searching can be shared among colleagues.

The Program Evidence Tool advocates the use of a broad range of evidence, including peer-reviewed literature, gray literature, content advisors, and informed colleagues and practice networks. Each category has strengths and weaknesses, and together the categories identify a broad range of evidence relevant to local programming.

Peer-reviewed literature includes publications indexed by major public vendors (eg, PubMed, Cochrane Collaboration, academic journals). With the proliferation of publications and the expense involved in keeping up with print and online journals, many practitioners see themselves as lost in the evidence rather than as being served by the evidence. As a practical solution, the Program Evidence Tool recommends many excellent and free sources of preappraised, synthesized, and web-based sources of evidence that address health promotion and chronic disease prevention (eg, Health-evidence.ca, *Guide to Community Preventive Services*, the Canadian Best Practices Portal).

Gray literature consists of publications (eg, government reports, conference proceedings, websites) that are not peer reviewed or indexed by major databases. Ideally, a public health librarian or an academic will be invited to provide guidance and feedback on the search strategy for gray literature; guidance becomes of greater importance when an evidence question concerns an issue for which little or no peer-reviewed evidence is available. Content advisors can be researchers with expertise in the topic area or community stakeholders who offer insights from lived experience (or both). Academic content advisors can provide feedback on the search strategy and access to unpublished research and generally help to fill gaps in the evidence.

**Figure 2:**
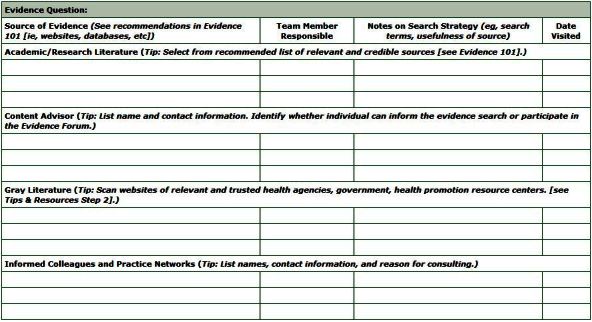
The search strategy worksheet documents the evidence sources to be searched in each of 4 evidence categories (eg, academic/research literature, content advisors, gray literature, informed colleagues/practice networks), assigns team members to search each source, and collates notes on the search process.

The search strategy worksheet (Figure 2) is used to document the sources of evidence to be searched within each category of evidence, the team member responsible for each site, date the sites are visited, and notes (eg, search terms used, whether or not useful sources of evidence were identified).

### Step 3: Collect and synthesize evidence

**Figure 3:**
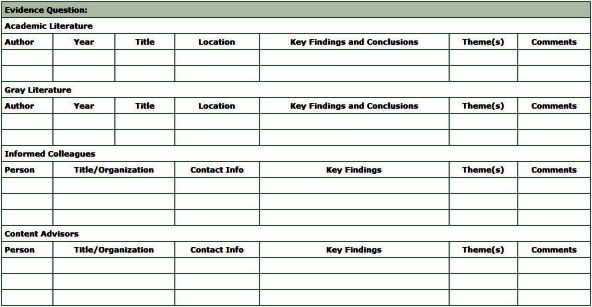
The evidence collection spreadsheet is a knowledge management tool to document the findings from the evidence searches.

The evidence collection spreadsheet (Figure 3) is a knowledge-management tool for organizing and documenting evidence findings. URL links to online evidence or paths to locally stored documents can be recorded on the spreadsheet to facilitate file retrieval. Once completed, evidence collection spreadsheets can be shared online to assist colleagues interested in searching a related evidence question. The time required to complete an evidence collection spreadsheet depends on the number of items being reviewed and the number of reviewers.

The evidence synthesis worksheet summarizes and synthesizes the main findings, questions raised by the evidence — or lack of evidence — and potential implications for practice. Synthesizing and summarizing large amounts of information is challenging. People with experience in qualitative research or identifying key themes are best suited to this task.

### Step 4: Interpret and adapt evidence

**Figure 4:**
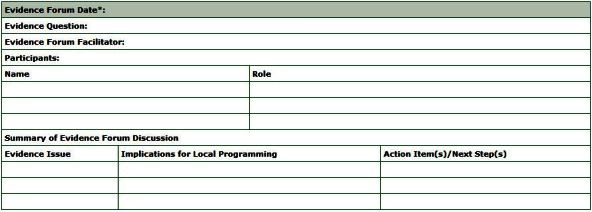
* All participants agree to review the evidence collection spread sheet and the evidence synthesis worksheet within 1 week of participating in the evidence forum. The evidence forum worksheet summarizes the evidence issues discussed, implications for local programming, including areas of consensus and/or disagreement and next steps agreed to during the evidence forum.

An evidence forum is a structured meeting among stakeholders to discuss the strengths of, gaps in, and implications of the evidence in local context. The evidence forum can occur in person or by teleconference. The main points from the forum discussion are recorded on the evidence forum worksheet (Figure 4). It is important to involve representatives from all levels (eg, upper management, front-line staff, content advisors, local stakeholders) in the discussion because all can benefit from this rich opportunity for knowledge exchange.

The objective of an evidence forum is to decide how to apply evidence findings. If current practice is consistent with evidence, little change may be required. If, however, the evidence suggests a significant departure from current practice, then local factors (eg, audience demographics and preferences, organizational and staff capacity, resources, political acceptability) must be considered. Challenges must also be overcome, such as evidence that is inconclusive or conflicting and feasibility of changing practice, including costs. Engaging a forum facilitator is recommended to ensure that all voices are heard and the discussion remains productive and reaches a timely conclusion.

The suggested timeline for an evidence forum is 90 to 120 minutes. A second evidence forum may be required if, for example, participants have not familiarized themselves with the evidence collection spread sheet and the evidence synthesis worksheet before meeting.

### Step 5: Implement and evaluate

**Figure 5:**
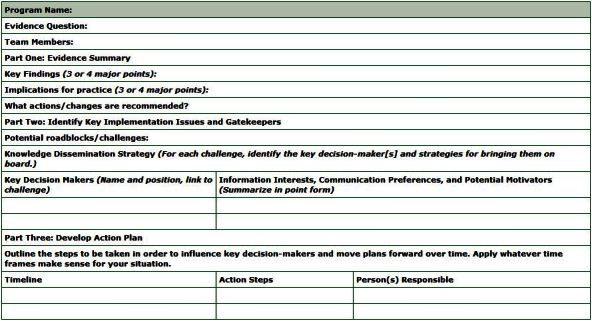
The knowledge dissemination strategy worksheet summarizes evidence findings and implications for practice into a few key points, assesses local barriers to applying the findings, shapes the message to match decision-makers’ information needs and presentation preferences, and documents an action plan to influence key decision makers and move plans forward.

Making the case for changing practice based on evidence can be facilitated by using effective knowledge dissemination strategies. These strategies summarize the evidence into a few key findings and implications for practice, shape the message to match users’ information needs and presentation preferences, capture users’ interest and inspire them to act on the changes, and monitor and support the change process. The knowledge dissemination strategy worksheet (Figure 5) guides the planning of a knowledge dissemination strategy and action plan.

Plans for change may unfold over months or even years, especially where resources are limited or resistance to change exists. In such cases, incremental change, beginning with the easier or more palatable changes, is a useful strategy. Finally, rather than assume that evidence-based change will have a positive impact, plans for process and outcome evaluation must be built in (25).

## Using the Program Evidence Tool

In 2008, the Program Evidence Tool was used to conduct 12 evidence searches by local community partnerships affiliated with 4 small to midsize regional public health departments. None were formally connected to academic institutions, and access to literature was limited to abstracts via PubMed, public-access journals, and online resources. One of the 4 organizations had support from an in-house librarian. Examples of initiatives for applying the tool included the following: a workplace wellness program, a school-based heart-health program, an initiative to link public health and municipal planners for the purpose of building healthy communities, and a nutrition education and cooking program for low-income women with young children.

The paragraphs below describe an example of using the Program Evidence Tool at a small, mainly rural public health department. The program selected for the evidence search was an initiative to provide a slow cooker and cooking classes featuring inexpensive slow-cooker recipes to low-income women with young children. The program was embraced by a public health nurse who wanted to address a need she saw in her community. A dietitian participated in the nutrition education aspect of the program. Health department management objected to the high cost of the program and wanted to discontinue it.

The evidence question originally proposed was “Is the use of slow cookers in a nutrition education and cooking class an effective way to improve the nutrition of low-income families with young children?” Working through Step 1, it became apparent that the focus of the original question was too narrow, and, after much discussion, the team realized they needed to address the broader issue of food security. The evidence question evolved to “What approaches are effective in building food security among low-income families with young children?” During the evidence search, the team found the most practical evidence in the gray literature. This literature included position papers on the effectiveness of community food security initiatives by Dietitians of Canada, publications from several health units that have been leaders in addressing local food security issues, and reports from the provincial and federal governments.

The content advisor selected by the team was a public health practitioner recognized as a leader in addressing food security in Ontario. She provided many useful sources of evidence and shared her experience of shifting the culture of her health department toward active engagement in local food security issues.

To obtain input from informed colleagues, a listserv request for information was disseminated across Ontario. Several useful responses detailed local experiences and reference lists. Because of inadequate local capacity, a researcher was hired to assist with completing the evidence collection worksheet and the evidence synthesis worksheet and with facilitating the evidence forum.

Participants at the evidence forum included the program manager, public health nurse, dietitian, content expert, consultant, and a representative from the Ontario Public Health Association. The group worked through seemingly contradictory evidence on whether community access to food security programs should be universal or targeted at the more marginalized subpopulations, opting for universal food delivery and targeted social support.

A major shift occurred in the group’s work when participants realized their organizational culture was part of the problem. The more they learned about food security through the evidence, the more they recognized the need for an organizational education initiative. They envisioned a 3-year plan, beginning with inviting guest speakers to educate their organization about food security. In the medium term, they planned to modify their programming to empower clients to become involved in program planning and to develop peer leaders. In the longer term, they planned to reach out to local social service agencies on joint community food security initiatives.

Use of the Program Evidence Tool transformed the vision of the participants and led to an ambitious plan to change the culture of their organization. Gray literature, content advisors, and informed colleagues were the most useful sources of evidence in this case. Management was impressed by the quality of the evidence collected and began to see links between the goals of community food security and requirements within the new Ontario Public Health Standards (1) to address the needs of people living in poverty.

## Benefits and Challenges

Using the Program Evidence Tool presented some challenges to the public health practitioners involved in the 4 health departments that tested the tools. Finding time to complete the steps was difficult because this work had to be completed in addition to handling a full workload of regular activities. Some groups spent several weeks settling on a final evidence question. Challenges were also in identifying and organizing the evidence. Much support was needed to do these tasks, mostly from a consultant or graduate student.

Organizational and contextual challenges were also evident. For example, community partnerships were challenged by insufficient time, limited organizational capacity and resources, and varied support from upper management in implementing evidence-informed recommendations. To overcome these challenges support for capacity building from the TEIP facilitator was provided. Strategies, such as train-the-trainer workshops, were developed subsequent to the pilot-testing phase, to address the identified need to build local capacity.

Public health practitioners using the Program Evidence Tool reported many benefits. These benefits were felt at the individual and program/organizational levels. At the individual level, reported outcomes included an increase in individual capacity development, especially as it pertained to knowledge and skills enhancement for the use of evidence. Users felt more competent in using research evidence and learned innovative approaches to health promotion and public health program planning. In addition, participants felt they gained awareness of and access to existing resources to support their work in public health. They recognized the benefit of connecting with a content expert and gained confidence in developing such a relationship.

At the program/organization level, many benefits for program planning and implementation were identified. Overall, participants felt they received increased support, especially from their managers, either in the expansion or in the continuation of their programs. Practitioners indicated that the Program Evidence Tool provided a way to review and systematically assess relevant evidence in their area of interest, which resulted in improved decision making and program planning.

## Discussion

Chronic disease prevention programs are influenced by various social, political, and structural factors. Evidence is needed that is not only valid and robust but also contextual and practical (16,26). The Program Evidence Tool supports use of a wide range of evidence sources, including randomized studies, needs assessments, local evaluations, and stakeholder opinion. Providing a tool through which varied sources of evidence can be considered allows for multiple viewpoints and empowers practitioners to make informed decisions about the programs they deliver.

Once the appropriate evidence has been identified, the next step is how to apply it in making informed decisions in public health practice. The quality of the evidence found must be considered, assumptions must be made explicit, and the decision-making process must be transparent (10). In the pilot test, users found that the Program Evidence Tool offered a capacity-building process to guide the selection and interpretation of evidence and promote an interactive process to enable “sense making” and appropriate use of evidence. The dialogue that resulted from the evidence forum was neither straightforward nor simplistic. Groups had to make decisions amid conflicting evidence, too much evidence, or not enough evidence. Much of the program-planning decisions could not be made on evidence alone, but rather on evidence combined with the knowledge and expertise of all stakeholders involved, promoting a 3-way dialogue between health promoters, researchers, and community stakeholders.

Public health practitioners must be skilled in identifying and applying relevant evidence to community-based programs to have a positive impact on the health of populations. The Program Evidence Tool provides a practical set of guidelines and worksheets to achieve this goal. The tool captures a wide range of evidence that is pertinent to practitioners. Use of the Tool facilitates an opportunity to debate and reflect on available evidence and contributes to program development that is supported and inspired by different forms of evidence and multiple viewpoints. The process acknowledges the expertise and experience of local chronic disease prevention practitioners in making decisions to improve their programs and the health of their communities.

## References

[1] Ontario Ministry of Health and Long-Term Care. Ontario public health standards 2008. Queens Printer for Ontario; 2008. http://www.health.gov.on.ca/english/providers/program/pubhealth/oph_standards/ophs/index.html. Accessed July 27, 2012.

[2] Core competencies for public health in Canada. Release 1.0. Public Health Agency of Canada; 2007. http://www.phac-aspc.gc.ca/php-psp/ccph-cesp/stmts-enon-eng.php. Accessed July 27, 2012.

[3] Council on Linkages Between Academia and Public Health Practice. Draft core competencies for public health professionals; 2008. http://www.phf.org/resourcestools/pages/core_public_health_competencies.aspx. Accessed July 27, 2012.

[4] Shilton T , Howat P , James R . Review of competencies for Australian health promotion. Promot Educ 2003;10(4):162–71. 15071971

[5] Franks AL , Brownson RC , Bryant C , Brown KM , Hooker SP , Pluto DM , Prevention research centers: contributions to updating the public health workforce through training. Prev Chronic Dis 2005;2(2):A26. 15888237 PMC1327720

[6] Centre for Evidence-based Medicine. Tools for each step of the EBM process. http://www.cebm.net/index.aspx?o=1023. Accessed: August 22, 2012.

[7] Hamilton S , McLaren S , Mulhall A . Assessing organisational readiness for change: use of diagnostic analysis prior to the implementation of a multidisciplinary assessment for acute stroke care. Implement Sci 2007;2:21. 10.1186/1748-5908-2-21 17629929 PMC1948015

[8] Skinner K . Developing a tool to measure knowledge exchange outcomes. Can J Program Eval 2007;22(1):49–73.

[9] Briss PA , Zaza S , Pappaioanou M , Fielding J , Wright-De Aguero L , Truman BI , . Developing an evidence-based guide to community preventive services — methods. The Task Force on Community Preventive Services. Am J Prev Med 2000;18(1 Suppl):35–43. 10806978 10.1016/s0749-3797(99)00119-1

[10] Tools to help organizations create, share and use research. Canadian Health Services Research Foundation. http://www.chsrf.ca/publicationsandresources/ResourcesForResearchers/SelfAssessmentTool.aspx. Accessed July 27, 2012.

[11] Kiefer L , Frank J , Di Ruggiero E , Dobbins M , Manuel D , Gully PR , Fostering evidence-based decision-making in Canada: examining the need for a Canadian population and Public Health Evidence Centre and Research Network. Can J Public Health 2005;96(3):I1–40 following 200. 15913085

[12] Rier DA , Indyk D . The rationale of interorganizational linkages to connect multiple sites of expertise, knowledge production, and knowledge transfer: an example from HIV/AIDS services for the inner city. Soc Work Health Care 2006;42(3–4):9–27. 16687372

[13] McNeil DA , Flynn MAT . Methods of defining best practice for population health approaches with obesity prevention as an example. Proc Nutr Soc 2006;65(4):403–11. 10.1079/PNS2006520 17181907

[14] Buffet C , Ciliska D , Thomas H . Can I use this evidence in my program decision? Assessing applicability and transferability of evidence. National Collaborating Centre for Methods and Tools; 2007. http://www.nccmt.ca/pubs/A&T_Tool_-_FINAL_English_Oct_07.pdf. Accessed August 22, 2012.

[15] National Cancer Institute Research-tested Interventions. Guidelines for choosing and adapting programs. http://rtips.cancer.gov/rtips/reference/adaptation_guidelines.pdf. Accessed August 22, 2012.

[16] Glasgow RE . What types of evidence are most needed to advance behavioral medicine? Ann Behav Med 2008;35(1):19–25. 18347901 10.1007/s12160-007-9008-5

[17] Last JM . Glossary of terms relevant to the core competencies for public health. Canada: Public Health Agency of Canada; 2007. http://www.phac-aspc.gc.ca/php-psp/ccph-cesp/glos-eng.php. Accessed July 27, 2012.

[18] Albert D , Fortin R , Lessio A , Herrera C , Riley B , Hanning R , Rush B . Strengthening chronic disease prevention programming: the Toward Evidence-Informed Practice (TEIP) program assessment tool. Prev Chronic Dis 2013;10:120106. 10.5888/pcd10.120106 PMC3675807 23721789

[19] Dobbins M , Ciliska D , Cockerill R , Barnsley J , DiCenso A . A framework for the dissemination and utilization of research for health-care policy and practice. Online J Knowl Synth Nurs 2002;9:7. 12439759

[20] DiCenso A , Guyatt G , Ciliska D . Evidence-based nursing: a guide to clinical practice. St. Louis (MO): Elsevier Mosby; 2005.

[21] Evidence-based practice: online course. Dietitians of Canada. http://www.dietitians.ca/Knowledge-Center/Events-and-Learning/Online-Courses/Evidence-Based-Decision-Making.aspx. Accessed July 27, 2012.

[22] Evidence guide: an introduction to finding, judging and using research findings on what works for children and young people. What Works for Children Group; 2003. http://www.whatworksforchildren.org.uk/docs/tools/evguide%20June6%20colour.pdf.Accessed July 27, 2012.

[23] Swinburn B , Gill T , Kumanyika S . Obesity prevention: a proposed framework for translating evidence into action. Obes Rev 2005;6(1):23–33. 10.1111/j.1467-789X.2005.00184.x 15655036

[24] Weighting up the evidence: making evidence-informed guidance accurate, achievable, and acceptable. Canadian Health Services Research Foundation; 2006. http://www.chsrf.ca/migrated/pdf/weighing_up_the_evidence_e.pdf. Accessed July 27, 2012.

[25] Quinn Patton M . Developmental evaluation: applying complexity concepts to enhance innovation and use. New York (NY): The Guilford Press; 2011.

[26] Graham ID , Logan J , Harrison MB , Straus SE , Tetroe J , Caswell W , Robinson N . Lost in knowledge translation: time for a map? J Contin Educ Health Prof 2006;26(1):13–24. 10.1002/chp.47 16557505

